# Body mass index, C-reactive protein, and pancreatic cancer: A Mendelian randomization analysis to investigate causal pathways

**DOI:** 10.3389/fonc.2023.1042567

**Published:** 2023-02-02

**Authors:** Zhenqi Li, Liquan Jin, Lu Xia, Xiangzhi Li, Yunfei Guan, Hongyang He

**Affiliations:** ^1^ School of Clinical Medicine, Dali University, Dali, China; ^2^ Department of General Surgery, The First Affiliated Hospital of Dali University, Dali, China; ^3^ College of Life Science, Shaanxi Normal University, Xi’an, China

**Keywords:** body mass index, C-reactive protein, pancreatic cancer, two-step Mendelian randomization, genetics

## Abstract

**Aim:**

To explore whether C-reactive protein (CRP) mediates the risk of body mass index (BMI) in pancreatic cancer (PC) and calculate the mediate proportion of CRP in this possible mechanism.

**Methods:**

Based on two-sample Mendelian randomization (TSMR), a two-step Mendelian randomization (TM) model was conducted to determine whether CRP was a mediator of the causal relationship between BMI and PC. The multivariable Mendelian randomization (MVMR) study was designed for mediating analysis and to calculate the mediating proportion mediated by CRP.

**Results:**

BMI has a positive causal relationship with PC (n = 393 SNPs, OR = 1.484, 95% CI: 1.021–2.157, p< 0.05). BMI has a positive causal relationship with CRP (n = 179 SNPs, OR = 1.393, 95% CI: 1.320–1.469, p< 0.05). CRP has a positive causal relationship with PC (n = 54 SNPs, OR = 1.348, 95% CI: 1.004–1.809, p*<* 0.05). After adjusting CRP, BMI has no causal relationship with PC (n = 334 SNPs, OR = 1.341, 95% CI: 0.884–2.037, p< 0.05). After adjusting BMI, there was still a positive causal relationship between CRP and PC (n = 334 SNPs, OR = 1.441, 95% CI: 1.064–1.950, p< 0.05). The mediating effect of CRP was 29%.

**Conclusions:**

In clinical practice, while actively advocating for weight loss among obese patients, we should focus on chronic inflammation levels in obese patients as well. In addition, anti-inflammatory dietary patterns and appropriate physical activity are important in preventing PC.

## 1 Introduction

Pancreatic cancer (PC), especially pancreatic ductal adenocarcinoma (PDAC), is the deadliest solid malignancy (1). The incidence and mortality rates of PC are increasing year by year, with little improvement in survival (2). PDAC is projected to become the second highest cause of cancer deaths in the United States by 2030 (3). It has been proven that developed countries carry a relatively higher attack rate of PC than developing countries (4). The incidence of PC is continuously increasing in China as well. It indicates that PC is closely related to diet and lifestyle (5). As the cornerstone of PC treatment, pancreaticoduodenectomy has undergone rapid technological development, but its overall prognosis and survival benefits for patients are not optimistic (6, 7). Therefore, maybe the prevention and screening of PC are more important than PC treatment (8).

The World Cancer Research Fund International and the American Institute of Cancer Research reviewed the impact of diet, nutrition, and physical exercise on PC, and believed that there was “strong evidence” that being overweight and obese would increase the risk of PC (9). One sign of obesity is adipose tissue inflammation, which can promote the growth of cancer by secreting proinflammatory cytokines (10, 11). This is supported by the paper by Jong Ho Park and others which indicated that chronic inflammation is associated with PC (12). Systemic low-grade inflammation could be a sign of obesity in adults, and adipose tissue is a key determinant of low-grade inflammation (13). The macrophages can seep into some adipose tissue and stimulate certain inflammatory factors that can create CRP in the liver (14, 15). Altogether, these findings suggest that systemic low-grade inflammation may act as a potential mediator of the relationship between obesity and PC, and the common marker of systemic inflammation includes CRP (16). Therefore, we infer that BMI has a positive causal relationship with PC through the mediation of CRP. However, the potential mediating effect of systemic low-grade inflammation on the association between obesity and specific symptom domains of pancreatic cancer remains uncertain.

Nevertheless, it should be noted that the observed associations could not be well determined due to the limitations of conventional statistical methods, namely, potential confounders of either or both reverse causalities. Mendelian randomization (MR) is a method that can be of support to resolve these limitations (17). In this research, we use BMI in place of obesity and use CRP instead of chronic inflammation in people. A two-step Mendelian randomization (TM) model analysis was performed to explore whether CRP mediates the risk of BMI on PC, and calculate the proportion of CRP in this possible mechanism.

## 2 Materials and methods

### 2.1 Data source and study design

Data on exposure variables for genetic variants associated with BMI were obtained from the IEU analysis of UK Biobank phenotypes (n = 461,460 participants) obtained through Genome-Wide Association Studies (GWAS) summary data (https://gwas.mrcieu.ac.uk/) on 11 July 2022 (individuals of European ancestry); similarly, data on outcome variables for PC were obtained from Finnish Biobank data obtained through GWAS, which included 605 PC cases and 218,187 controls of European ancestry. CRP data were obtained from the International HapMap Project (HapMap), including 204,402 samples (18). Brief information is shown in **Table 1**.

**Table 1:** Summary of the GWAS included in this MR study neoplasms.

Exposures/outcomes	GWAS ID	Consortium	Ethnicity	Sample sizes	Number of SNPs	Sex	Year
BMI	ukb-b-19953	UKB	European	461,460	9,851,867	Male and Female	2018
Malignant neoplasm of pancreas	finn-b-C3_PANCREAS	FB	European	218,792 (ncase: 605; ncontrol: 218,187)	16,380,466	Male and Female	2021
C-reactive protein level	ieu-b-35	HapMap	European	204,402	2,414,379	Male and Female	2018

**Figure 1:**
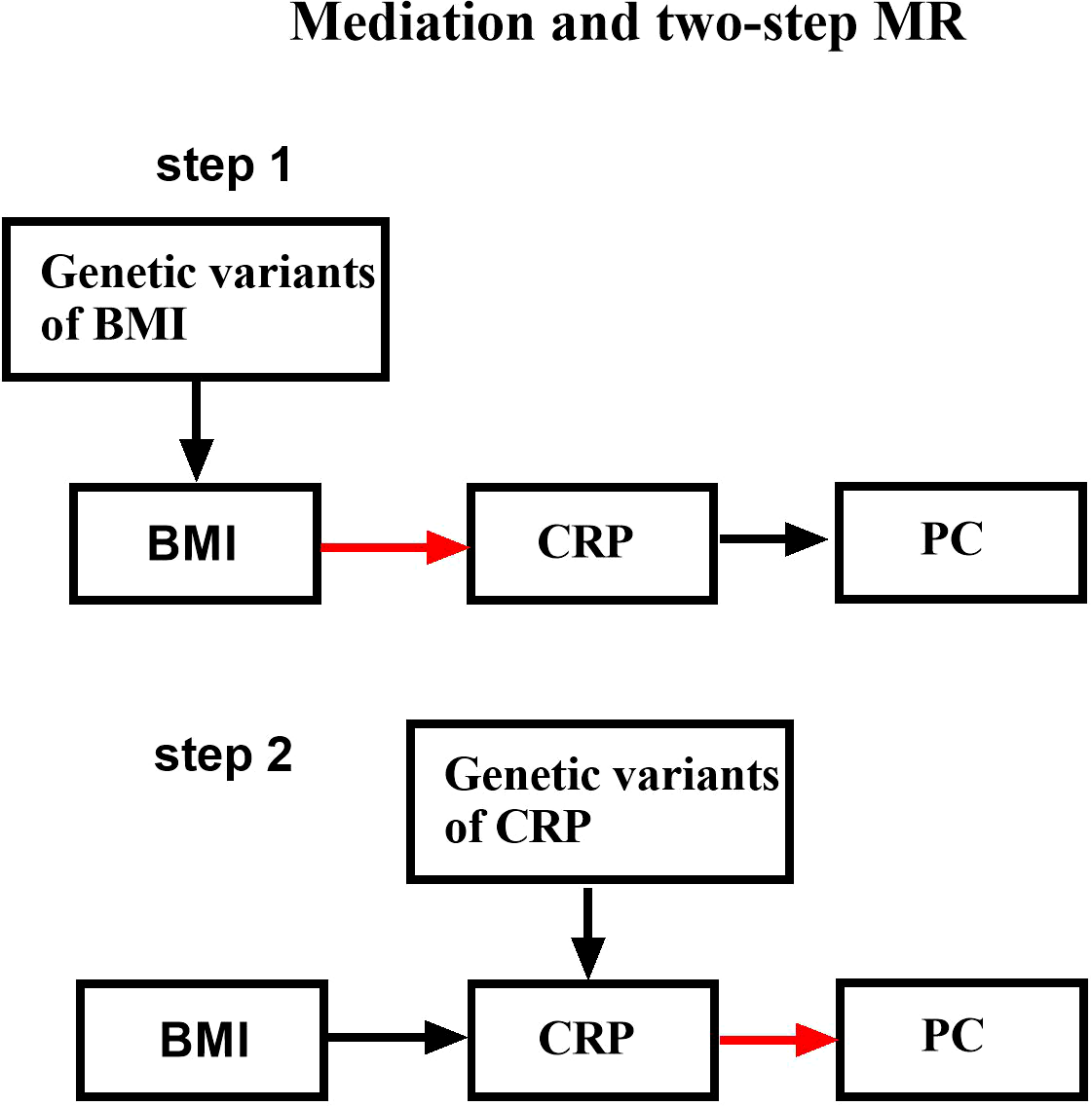
Schematic design showing the study process (hypothesis 1: significantly correlated with exposure; hypothesis 2: not correlated with outcome; hypothesis 3: not correlated with confounders).

In total, our research model was carried out in two steps (**Figure 1**). Firstly, two-sample MR (TSMR) was performed to identify exposure variables as BMI and outcome variables as PC or CRP. Secondly, TM was performed to identify exposure variables as CRP and outcome variables as PC by searching open genome-wide association study GWAS data. A two-step Mendelian randomization (TM) model was conducted to determine whether CRP was a mediator of the causal relationship between BMI and PC. The multivariable Mendelian randomization (MVMR) study aims to calculate the mediating proportion mediated by CRP.

### 2.2 Selection of genetic instrumental variables

Genetic variants used in the MR analyses were of genome-wide significance (p< 5×10^−8^) and were distributed independently by pruning SNPs with an *r^2^* < 0.001 threshold. The phenotypes related to the remaining SNPs were searched through the human genotype-phenotype (19) association database, and the widely accepted SNPs related to affect outcome variables were excluded. The information of the outcome was extracted by GWAS, and the relationship between the SNPs and the outcome satisfying assumptions 1, 2, and 3 was obtained from the outcome. The exposure and outcome datasets were combined, which included the relationship between the above instrumental variables and outcomes and exposures, and removed incompatible alleles and palindromic SNPs. The remaining SNPs were the final instrumental variables exposed.

### 2.3 Mendelian randomization analyses

The inverse variance weighted (IVW) random-effects model (20), IVW fixed-effects model (20), and robust adjusted profile scores (RAPS) method (21) were applied to perform a TSMR analysis. In addition, the odds ratios and 95% confidence intervals (CIs) for the three approaches were presented. IVW improves the estimation accuracy and testing capabilities. The RAPS method, which does not consider horizontal pleiotropy or outliers, is a relatively recent approach.

### 2.4 Sensitivity analyses

In the first and two steps, the IVW random-effects model, the IVW fixed-effects model, and the RAPS method were used to perform a TSMR analysis, and the Cochran Q test was used to evaluate the heterogeneity between individual genetic variation estimates. If p< 0.05 for Cochran’s Q-test (22), the final results of MR were referred to the IVW random-effects model; otherwise, a fixed-effects model was used. In order to check whether there was a violation of the MR assumption due to horizontal pleiotropy, egger-level pleiotropy was performed — the intercept method, in which the cutoff value estimates whether the genetic variation significantly affects the outcome through a route other than exposure. Finally, the Radio package is used to identify the real outliers. If there are outliers, the MRPRESSO method was used to evaluate the effect of the outliers on the results (23).

### 2.5 Count the mediation proportion

The TSMR method was used to obtain the *β*1 value from BMI to CRP; the *β*2 value from CRP to PC was obtained, and then *β*3 value from BMI to PC was obtained, paying attention to whether the mechanism could be passed through. The multivariate Mendelian randomization (MVMR) method was then used to determine the association between the remaining exposure and the outcome PC after adjusting for CRP or BMI. The *β* value of CRP to PC after BMI control is *β*4 ; The *β* value of BMI to PC after CRP control is *β*5 . Use “mediation proportion = $\frac{\beta 1*\beta 4}{\beta 1*\beta 4+\beta 5}$ " to calculate the mediation proportion of CRP (24). "*β*1*β4" represented the indirect effect of the mediator in the mechanism. "*β*5" represented the direct effect of the mediator in the mechanism. "*β*1*β4+*β*5" represented the total effect of the mediator in the mechanism. All the above methodologies and visualization graphs were obtained using R version 4.1.2 and GraphPad Prism 9.0.

### 2.6 Evaluation of genetic instrumental variables

*R* ^2^ and *F*-value were calculated to use the calculation formula: *R* ^2^ = 2x (1-*MAF*) (*MAF*) ( $\frac{\beta }{SD}$ )^2^, where MAF is the minor allele frequency of exposure, *β*  is the allele effect value of exposure, and *SD* is the standard deviation. Note that *R* ^2^ is the sum of the *R* ^2^ of all SNPs. Reuse the calculation formula: *F* = $\frac{R^{2}\left(\text{N}-\text{K}-1\right)}{\left(1-R^{2}\right)\text{K}}$ Among them, N is the total number of exposed samples, K is the number of SNPs, and *R* ^2^ is the same as above. The calculated *F* value greater than 10 indicated a strong instrumental variable, while less than 10 indicated a weak instrumental variable (25).

## 3 Results

The remaining 458 SNPs of BMI-related genetic variation that simultaneously meet assumptions 1, 2, and 3 were screened from the human genotype–phenotype association database; meanwhile, the SNPs related to smoking (26) (rs10927006, rs3845344, rs12089815, rs2568958, rs34517439, rs6545714, rs10182416, rs9876664, rs78605811, rs9835772, rs1454687, rs75499503, rs74750282, rs3901286, rs215634, rs12541408, rs7024334, rs11012732, rs6265, rs317656, rs7132908, rs9515446, rs862320, rs9926784, and rs35154326) were deleted. As the aforementioned 25 SNPs were removed, only 433 were retained. Information on PC was extracted by GWAS, and the relationship between the above 433 SNPs and the outcome was determined by analyzing the study outcomes. As the FB was unable to determine 16 RS loci in PC, 417 SNPs remained. Hence, the exposure and outcome dataset were merged, which showed the relationship between the 417 tool variables and the outcomes and exposures, and palindrome SNPs (rs10832778, rs10887578, rs11250094, rs11634851, rs12507026, rs1608113, rs1860750, rs2253310, rs2396625, rs2618039, rs347551, rs355777, rs396755, rs4419475, rs4737188, rs59086897, rs6597975, rs7568228, rs765874, rs770482, rs9388446, and rs961498) and incompatible allele (rs7928320 and rs9674487) were deleted. The 393 instrumental variables were considered as the final instrumental variables referring to BMI (BMI to PC).

Similarly, the last remaining 54 instrumental variables are the final instrumental variables referring to CRP (CRP to PC). The remaining 57 SNPs of BMI-related genetic variation that simultaneously meet assumptions 1, 2, and 3 were screened from the human genotype–phenotype association database; meanwhile, the SNPs related to smoking (n = 0) were deleted. The FB was unable to determine 1 RS loci in PC. The palindrome SNPs (rs10778215 and rs11108056) were deleted.

The remaining 458 SNPs of BMI-related genetic variation that simultaneously meet assumptions 1, 2, and 3 were screened from the human genotype–phenotype association database; meanwhile, the SNPs related to cancer (rs34517439, rs10169594, rs1229984, rs11012732, and rs961498) and to inflammatory disease (rs1064213, rs9843653, rs62407562, rs28366156, rs9267671, and rs11362410) were deleted. As the aforementioned 11 SNPs were removed, only 447 were retained. Information on CRP was extracted by GWAS, and the relationship between the above 447 SNPs and the outcome was determined by analyzing the study outcomes. As the FB was unable to determine 258 RS loci in PC, 189 SNPs remained. Hence, the exposure and outcome dataset were merged, which showed the relationship between the 189 tool variables and the outcomes and exposures, and palindrome SNPs (rs7928320) and the incompatible allele (rs10887578; rs11250094; rs1454687; rs2396625; rs396755; rs4737188; rs7568228; rs765874 and rs9388446) were deleted. The last remaining 179 instrumental variables are the final instrumental variables referring to BMI (BMI to CRP).

**Figure 2:**
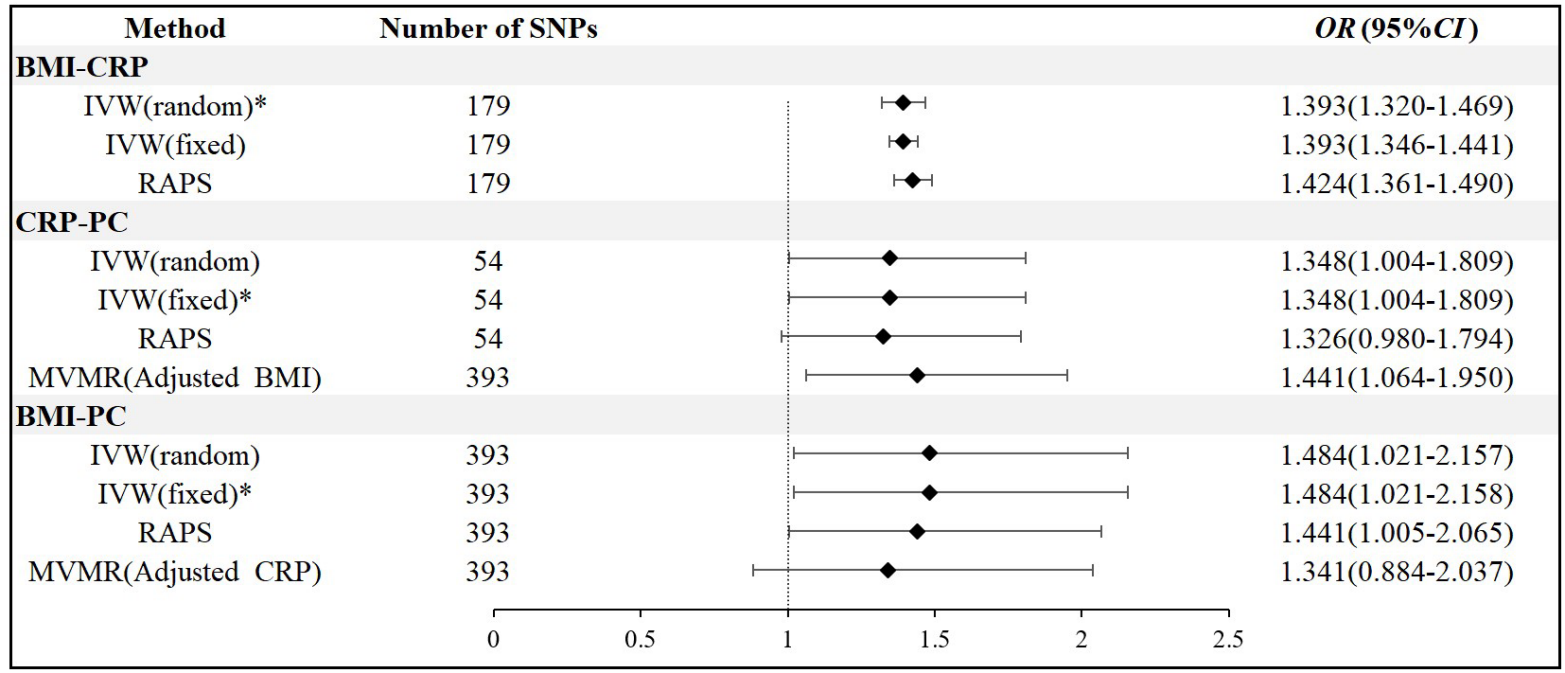
Two-sample and multivariable Mendelian randomization results.

**Figure 3:**
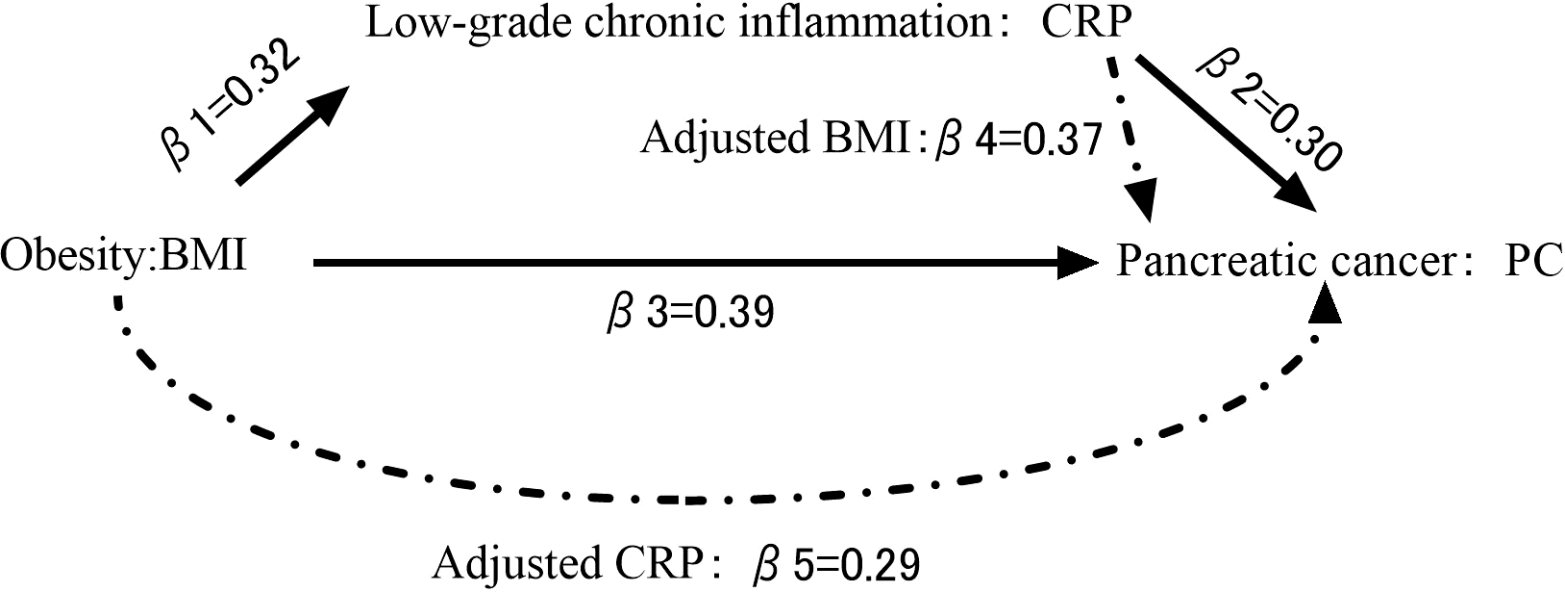
Diagram of causal path.

There was a positive causal relationship between BMI and PC, and the IVW fixed-effects model was used as the gold standard (n = 393 SNPs, OR = 1.484, 95% CI: 1.021–2.157, p< 0.05), *β*3 = 0.39. There was a positive causal relationship between BMI and CRP, and the IVW random-effects model was used as the gold standard (n = 179 SNPs, OR = 1.393, 95% CI: 1.320–1.469, p< 0.05), *β*1 = 0.32. As mentioned above, the IVW fixed-effects model was used as the gold standard for CRP and PC, and there was a positive causal relationship (n = 54 SNPs, OR = 1.348, 95% CI: 1.004–1.809, p< 0.05), *β*2 value = 0.30. After controlling for CRP, there was no causal relationship between BMI and PC (n = 334 SNPs, OR = 1.341, 95% CI: 0.884–2.037, p > 0.05), *β*5  = 0.30. After controlling for BMI, there was still a positive causal relationship between CRP and PC (n = 334 SNPs, OR = 1.441, 95% CI: 1.064–1.950, p< 0.05), *β*4  = 0.37. Therefore, the mediation effect of CRP is 29% (**Figures 2**, **3**).

The IVW approach computed the heterogeneity of MR results from BMI to CRP (p = 3.19E-23), showing obvious heterogeneity. The egger intercept approach yielded the level pleiotropy test findings (p = 0.82), indicating that the instrumental variables did not significantly affect the outcome (CRP) in other ways than exposure (BMI). The IVW random model was used as the gold standard (**Table 2**). Using radio to identify the outliers, it is feasible to demonstrate that if BMI is the exposure variable and CRP variable is the outcome variable, there are ten outliers (rs10423928, rs11250094, rs11610621, rs13107325, rs17056301, rs2606228, rs4261944, rs4658403, rs4764949, and rs7519259) among the 179 tool variables screened. After using the MRPRESSO method for verification, the initial p value was 8.53E-27; after correction and elimination of the ten outliers, the p value was 2.71E-39. The outliers had a minimal impact on the results, demonstrating that the study findings are resilient (**Table 2**).

**Table 2:** The results of the heterogeneity and horizontal pleiotropy.

Causal path	SNPs	Cochran’s Q heterogeneity test	MR Egger pleiotropy test *P* value	Outliers (Yes/No)	*P* Value before MRPRESSO correction	After correction and elimination
		*P* value (IVW)				
BMI to CRP	179	3.19E-23	0.82	Yes	8.53E-27	2.71E-39
CRP to PC	54	0.94	0.44	No	–	-_
BMI to PC	393	0.80	0.15	No	-_	-_

The heterogeneity of MR results from CRP levels to PC calculated by the IVW method was p = 0.94, indicating no noticeable heterogeneity. The egger intercept approach yielded the level pleiotropy test findings (p = 0.44), indicating that the instrumental variables did not significantly affect the outcome (PC) in other ways than exposure (CRP). The IVW fixed model was used as the gold standard. However, with CRP as the exposure variable and PC as the outcome variable, no outliers were found in the selected 54 instrumental variables (**Table 2**).

The heterogeneity of MR results from serum BMI to PC calculated by the IVW method was p = 0.80, indicating no noticeable heterogeneity. The egger intercept approach yielded the level pleiotropy test findings (p = 0.15), indicating that the instrumental variables did not affect the outcome (PC) in other ways than exposure (BMI). The IVW fixed model was used as the gold standard. Similarly, with CRP as the exposure variable and PC as the outcome variable, no outliers were found in the selected 393 instrumental variables (**Table 2**).

Finally, using the formulas for *R* ^2^ and *F* value, we calculated that the *F* value of the instrumental variable from BMI to CRP to refer to BMI was 25, the *F* value of BMI to PC to refer to BMI was 24, and the *F* value of CRP to PC to refer to BMI was 24 and the *F* value of the instrumental variable for BMI was 78. All are greater than 10, which proves that the TSMR in this study refers to the instrumental variables of exposure are all strong instrumental variables.

## 4 Discussion

In this two-step MR study, we found that CRP mediated the important significance of BMI on the occurrence of PC. After controlling for CRP, there was no causal relationship between BMI and PC. The mediation effect of CRP is 29%.

Our study pays tribute to Carol Barahona Ponce et al.’s paper on the causal relationship between gallstones, BMI, and CRP in gallbladder cancer (27). We selected CRP as a marker of chronic inflammation in the human body to explore its mediating role in obesity-induced PC. After reviewing a large body of data, we are concerned that chronic inflammation is an important risk factor for PC (28). However, in recent years, the research direction has begun to shift to the value of inflammatory markers in the human body on the survival and prognosis of PC. Few researchers have paid attention to the fact that chronic inflammation itself is a critical factor in the occurrence of PC (29). In addition, as obesity, which is a global issue, has caused a huge change in the human disease spectrum, the relationship between blood lipids, inflammation, obesity, and cancer has attracted sufficient attention. Although in recent years, researchers have discovered that obesity is also an important risk factor for PC through observational research, doubts regarding the causal relationship between obesity and pancreatic cancer remain due to the limitations of observational research. Interestingly, some scholars have confirmed the positive causal relationship between obesity and pancreatic cancer by MR, which is consistent with our findings (30). Nevertheless, we need to emphasize an MR study published by the GUT in 2021, whose author disclosed that obesity may play an important mediating role in diabetes causing PC (31). In this regard, our research is designed based on this information to try to establish a more detailed mechanism, that is, to emphasize the mediating role of chronic low-grade inflammation in the relationship between obesity and PC. From an isolated and one-sided perspective, the relationship between chronic low-grade inflammation, obesity, and pancreatic cancer seems to be confirmed. However, it is difficult to demonstrate the complete causal chain through MR. PC has strong geographical variability and dietary variability. At this point, our TM was implemented based on different sample databases of European races. It utilizes genetic data to avoid confounding and reverse causality, and our sensitivity analysis proves that the horizontal pleiotropy test of any causal path is not significant. This enables us to provide sufficient credibility for the demonstration of this mechanism. In addition, a high-quality cohort study in 2020 can support our conclusion that CRP increase is positively related to PC risk, but it is only significant in patients with metabolic syndromes (16).

Our study confirmed the linkage mechanism that obesity contributes to the development of PC by inducing chronic inflammation, in which we established through MVMR that once chronic inflammation is controlled, the effect of BMI on PC has no significance. A recent study reveals that an inflammatory factor axis mediates neuroinvasion in PC (32). Hence, inflammatory factors are of great significance for the occurrence, development, prognosis, and survival of PC. In addition to CRP, interleukin-18, TPX3, interleukin (IL)-33, etc., have potential for further exploration as well (12, 33, 34).

Through the realization of this mechanism and causal path, we may reduce the incidence of pancreatic cancer by intervening in any of the links. For example, an increasing number of studies have shown a positive relationship between pro-inflammatory diets such as the consumption of red meat, processed meat, high-fat diet, etc., and PC (35). Based on our MR results, we demonstrated the importance of chronic inflammation in the occurrence of PC and proved that a pro-inflammatory diet may have an important promoting effect on the occurrence of PC. People on long-term pro-inflammatory diets have chronic inflammation, which supports previous observational studies. For example, fish oil is an Omega-3-rich health supplement that is popular around the world for its efficacy in preventing PC (36). From our research, this appears to be true. An anti-inflammatory diet includes increased consumption of Omega-3 unsaturated fatty acids, polyphenolic compounds, dietary fiber, etc. (37). Our study provides a basis for an anti-inflammatory diet to prevent PC, and for studies where physical activity, anti-inflammatory dietary patterns such as the Mediterranean diet and lifestyle, and even bariatric surgery are recommended to prevent PC (38–40). Obese patients should improve their eating patterns and incorporate weight loss and physical exercise into their daily routines. Clinically, doctors should pay more attention to the level of inflammation in obese patients.

There are various limitations in our study. Firstly, our dataset only includes European populations, which limits the applicability of the results to non-European populations. Therefore, further research is required to verify the applicability of these results to other ethnicities. Second, BMI to CRP MR was heterogeneous. Although a random-effects model was used at the end to give robustness to the results, the heterogeneity has an impact on the accuracy of the calculated value of the CRP intermediary ratio. Third, our MR is only linear. In the future, a larger prospective cohort study is required to replicate our analysis.

Nonetheless, this study has proven the causal relationship between BMI, CRP, and PC to some extent, and demonstrated the importance of CRP-mediated BMI and PC. This study is significant in guiding decision-making in our clinical work and the diet and lifestyle of the population.

## 5 Conclusion

In clinical practice, while actively advocating for weight loss among obese patients, we should focus on chronic inflammation levels in obese patients as well. In addition, anti-inflammatory dietary patterns and appropriate physical activity are important in preventing PC.

## Data availability statement

The datasets presented in this study can be found in online repositories. The names of the repository/repositories and accession number(s) can be found in the article/**Supplementary Material**.

## Ethics statement

This study is based on public database. Ethical review and approval was not required for the study on human participants on public database in accordance with the local legislation and institutional requirements. Written informed consent for participation was not required for this study in accordance with the national legislation and the institutional requirements.

## Author contributions

ZL wrote the manuscript and performed the quality assessment. LJ designed the project and performed the statistical analysis. YG and HH contributed to the revision of the manuscript and reviewed the results. Conceptualization, ZL and LX; methodology, YG; software, ZL; validation, ZL; formal analysis, XL; investigation, ZL; resources, ZL; data curation, ZL; writing—original draft preparation, ZL; writing—review and editing, ZL; visualization, ZL; supervision, HH; funding acquisition, LJ. All authors contributed to the article and approved the submitted version.
