# Supplementary figures and images for: Body mass index, C-reactive protein, and pancreatic cancer: A Mendelian randomization analysis to investigate causal pathways
Source: Front Oncol. 2023 Feb 2;13:1042567. doi: 10.3389/fonc.2023.1042567 (PMC9932924; doi:10.3389/fonc.2023.1042567)

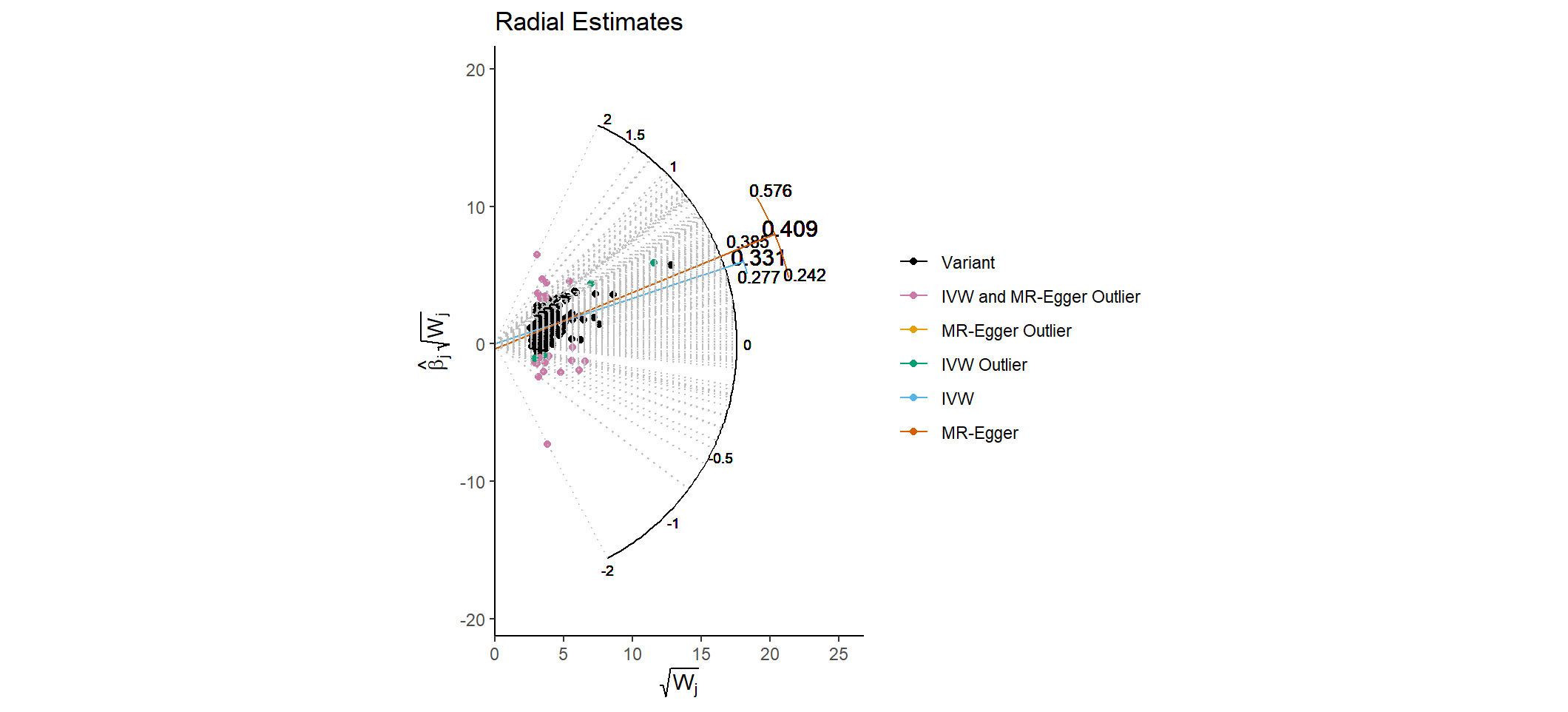

Supplement: Supplementary file 1 [file DataSheet_1.zip › Image 1.PNG]

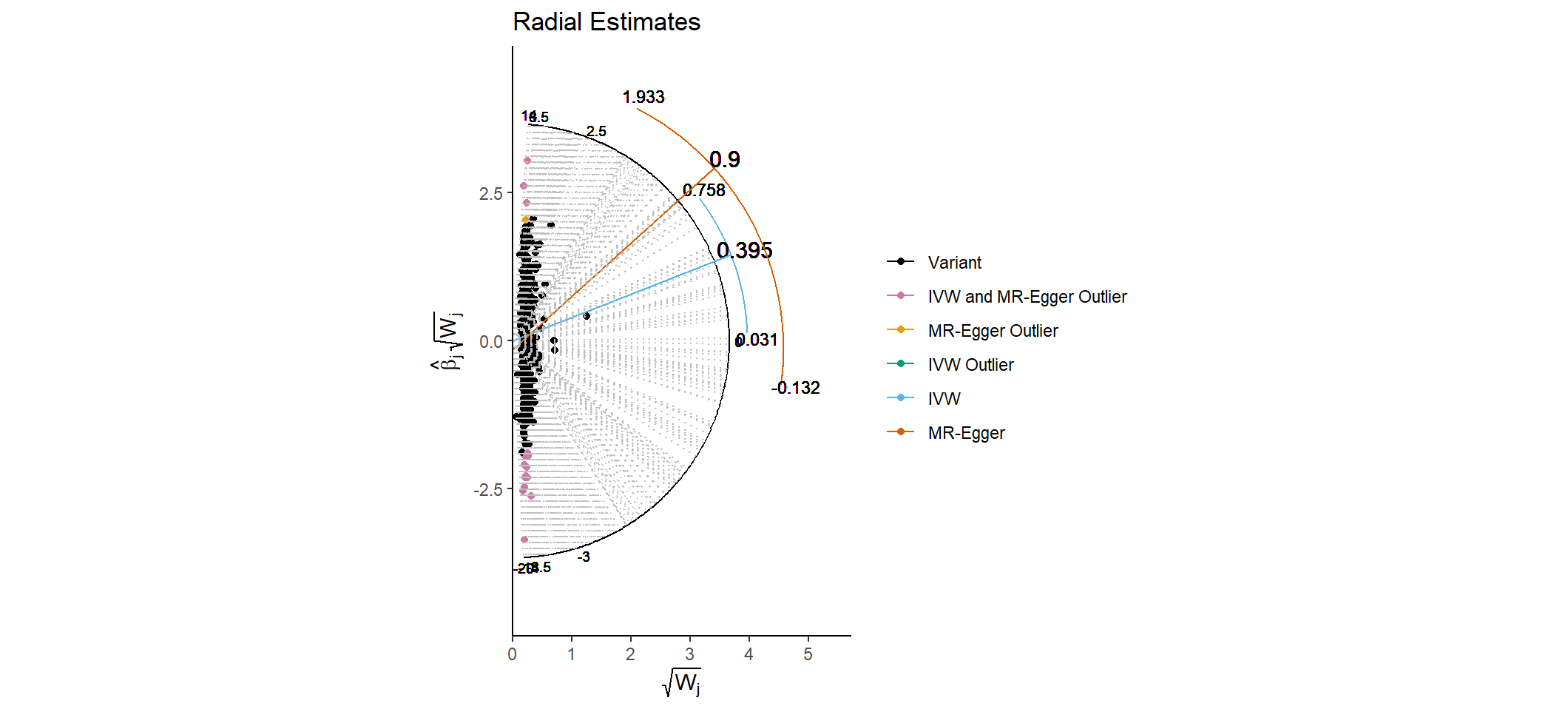

Supplement: Supplementary file 1 [file DataSheet_1.zip › Image 10.PNG]

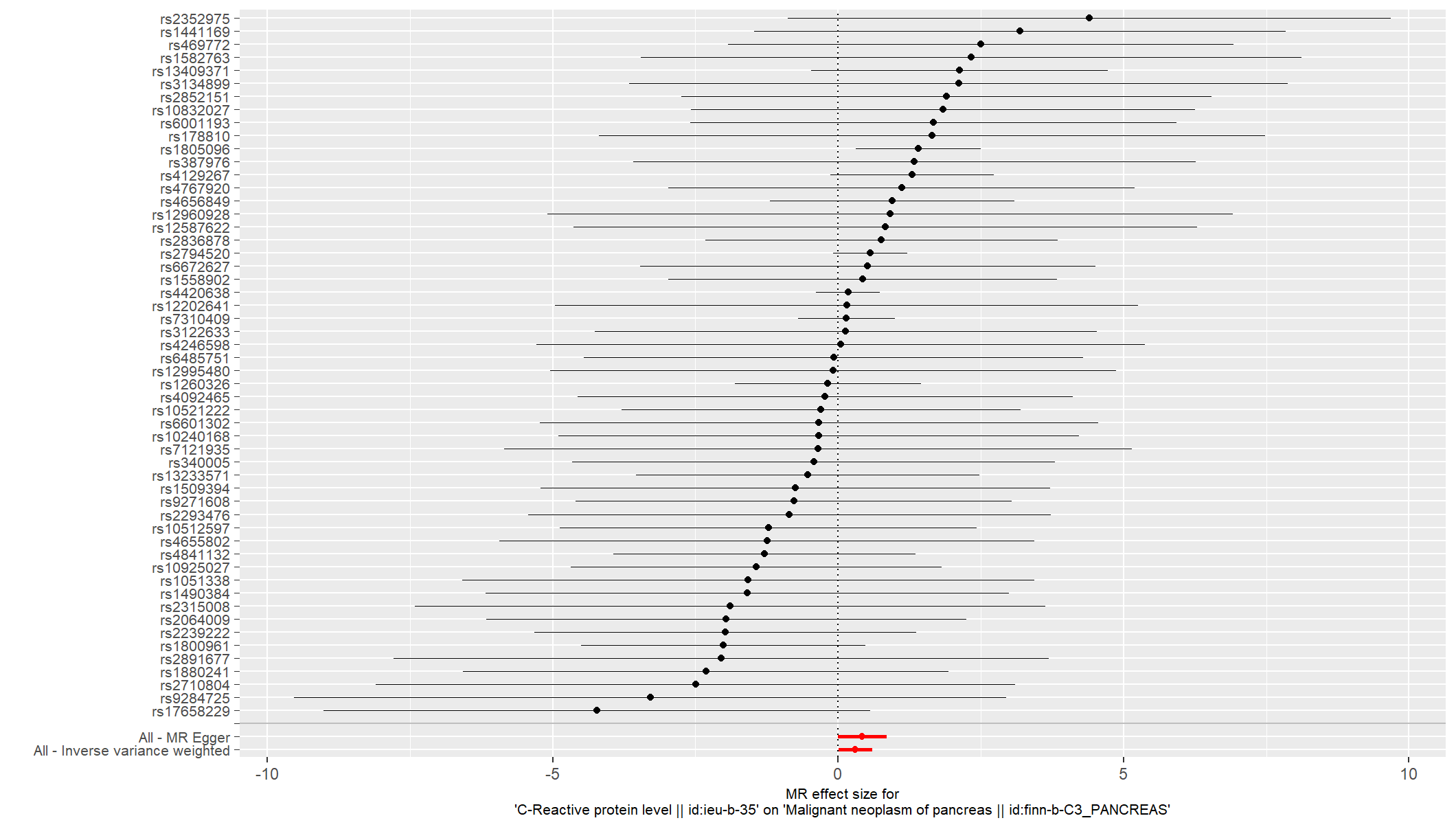

Supplement: Supplementary file 1 [file DataSheet_1.zip › Image 11.PNG]

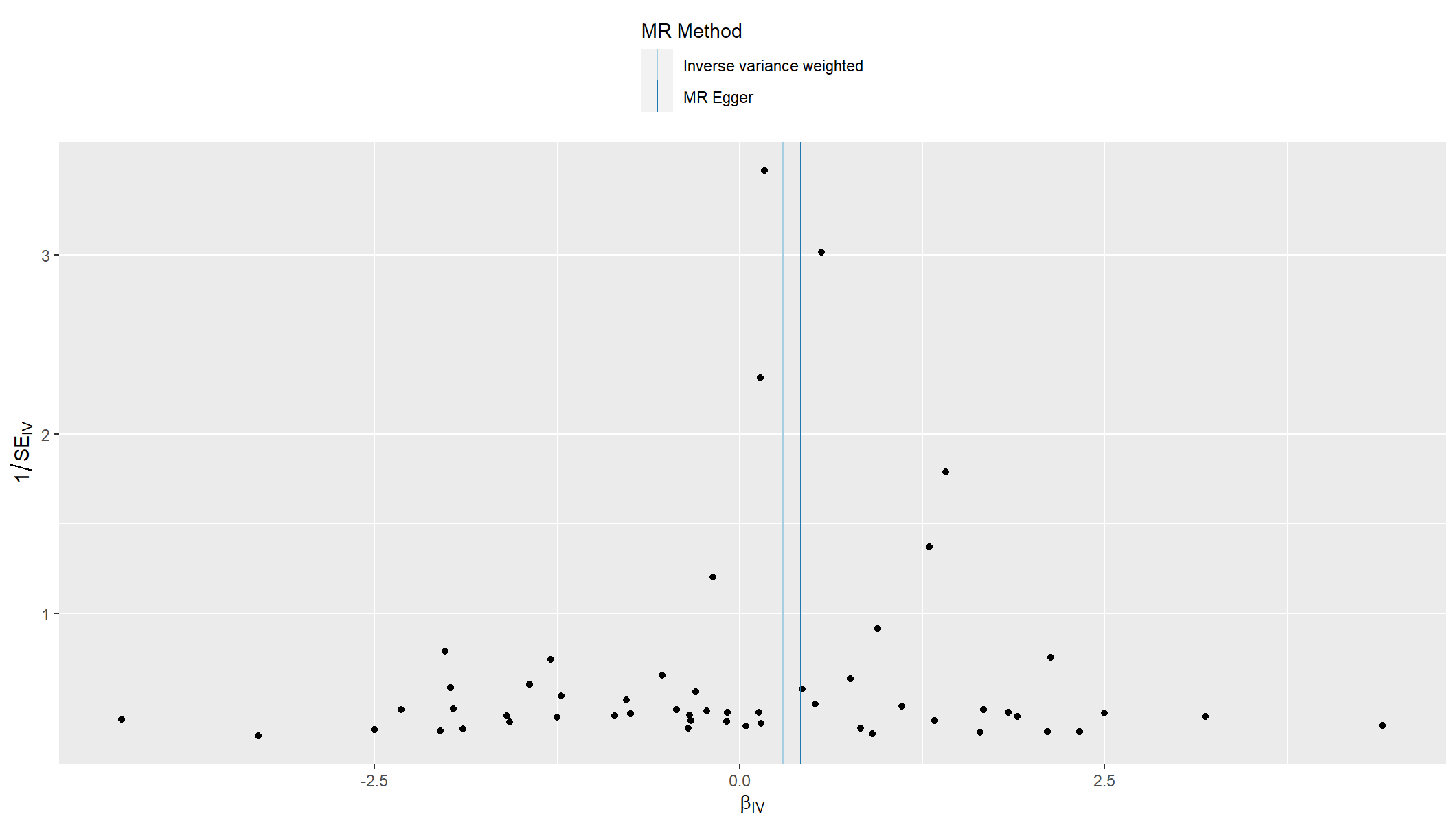

Supplement: Supplementary file 1 [file DataSheet_1.zip › Image 12.PNG]

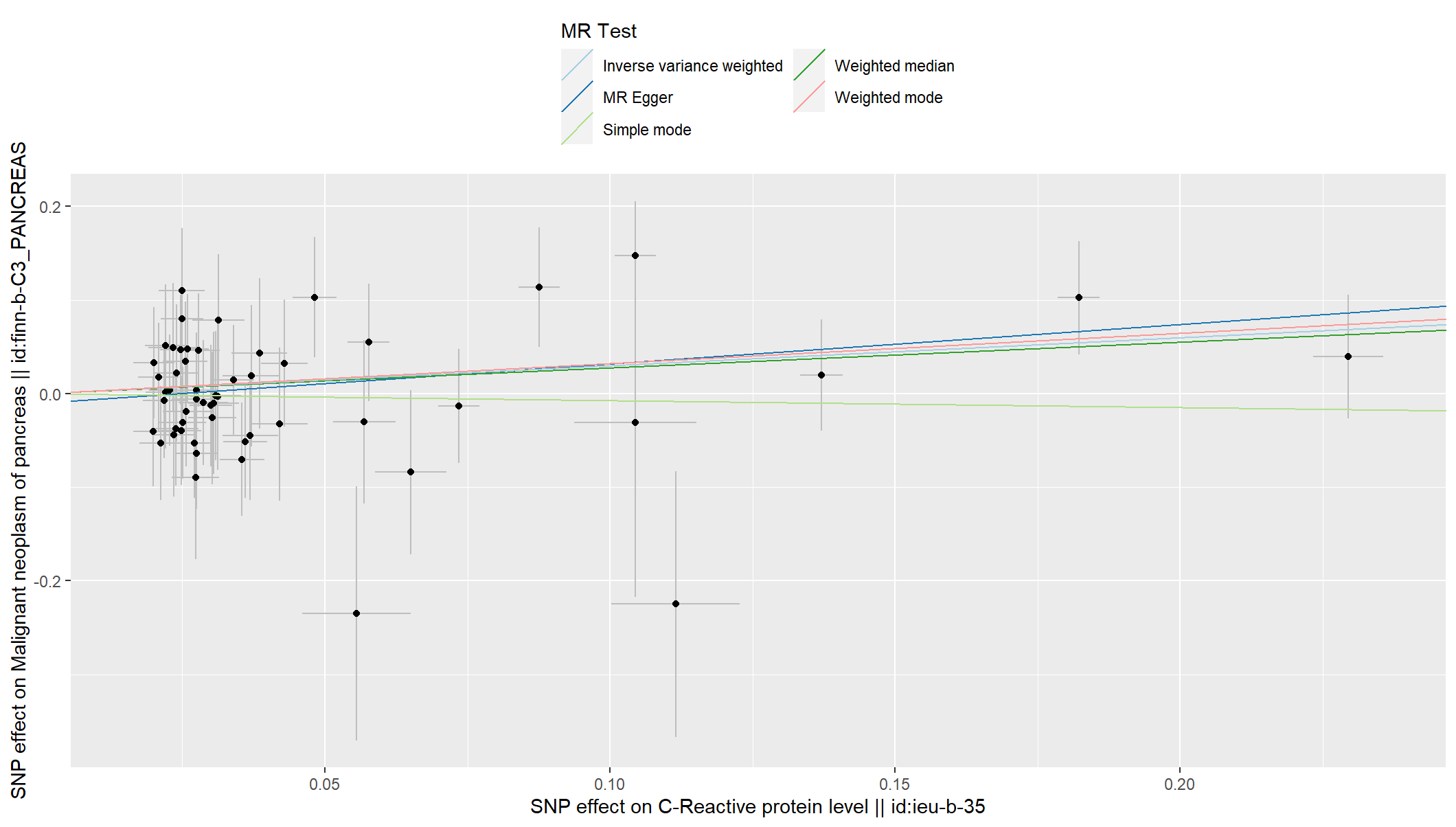

Supplement: Supplementary file 1 [file DataSheet_1.zip › Image 13.PNG]

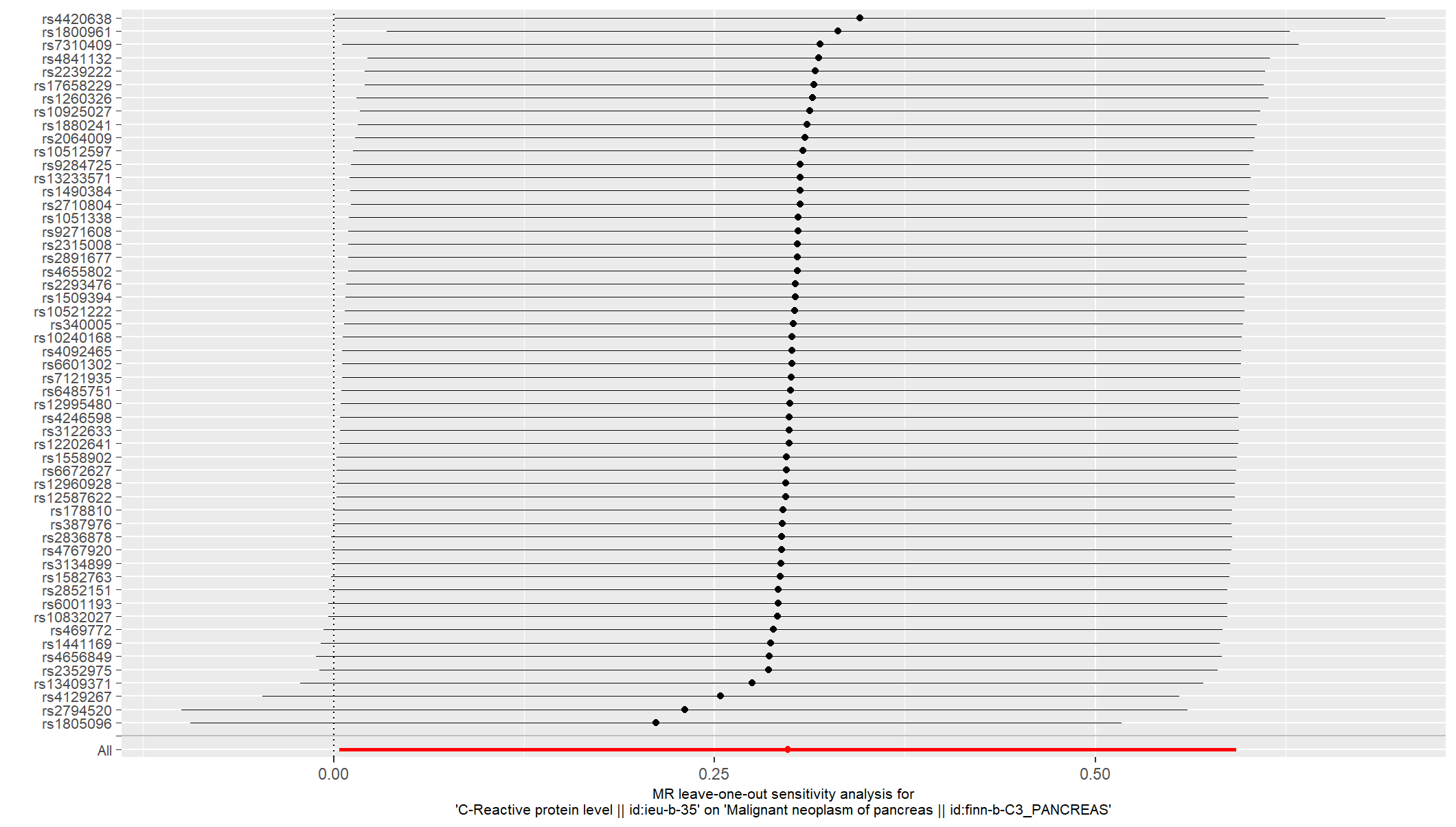

Supplement: Supplementary file 1 [file DataSheet_1.zip › Image 14.PNG]

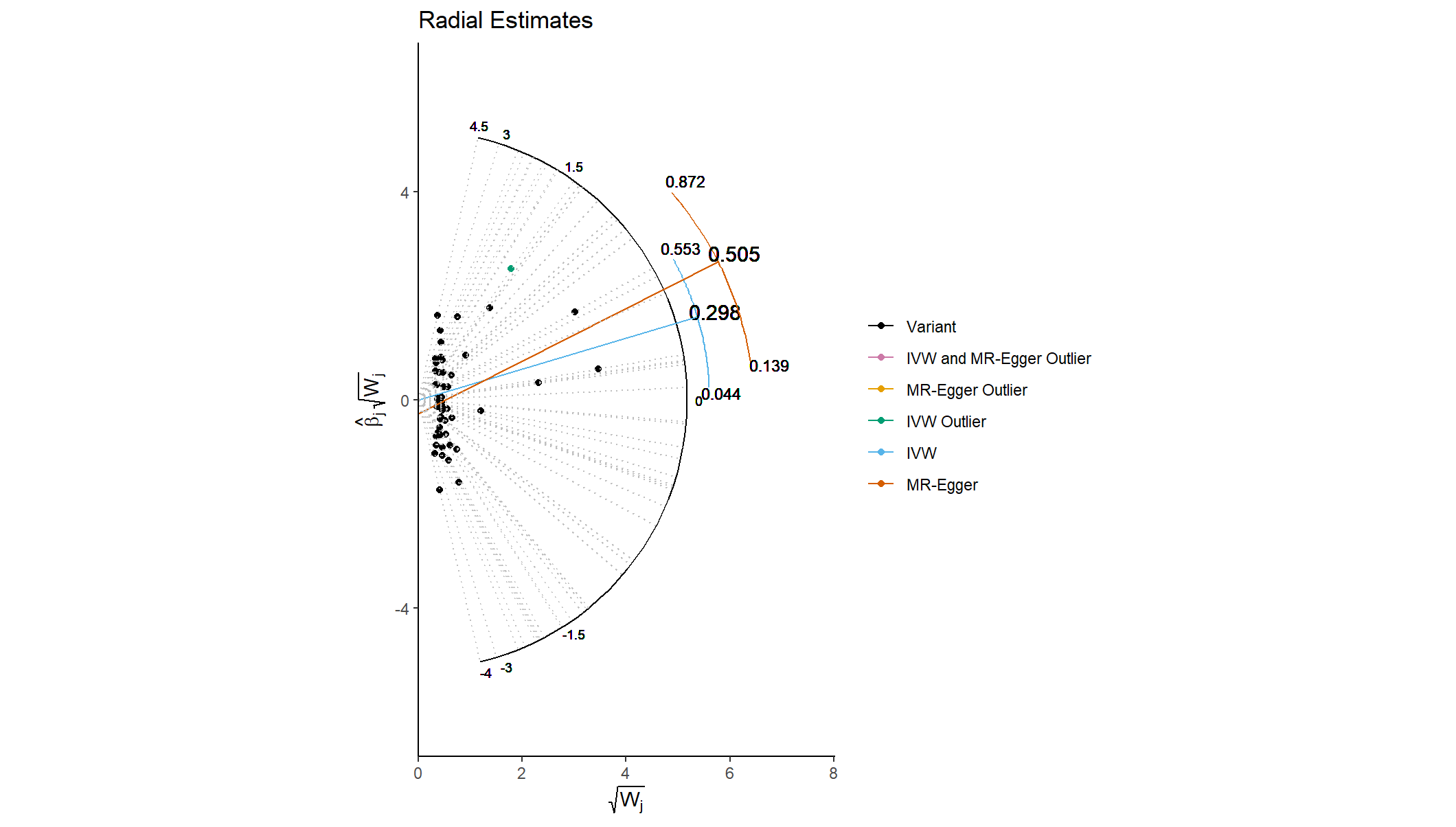

Supplement: Supplementary file 1 [file DataSheet_1.zip › Image 15.PNG]

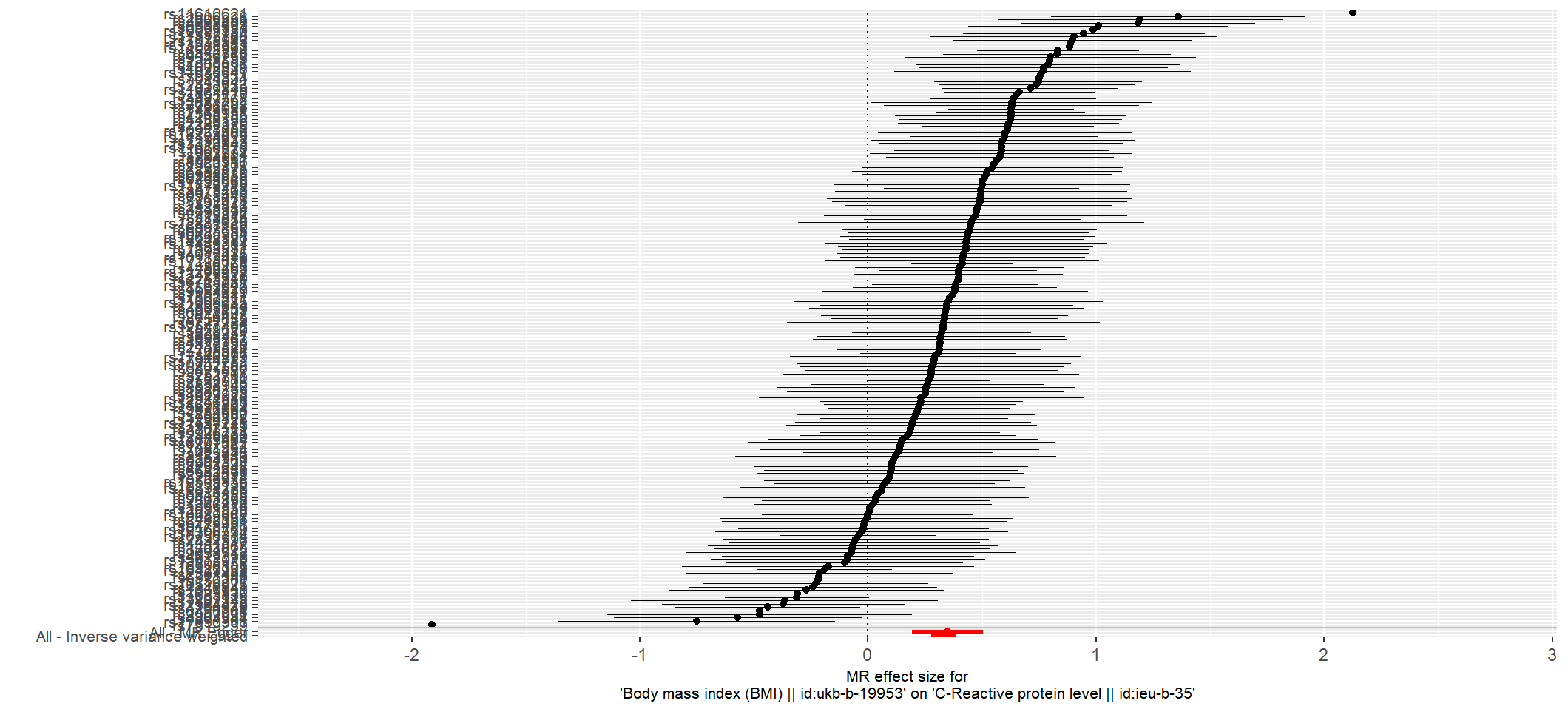

Supplement: Supplementary file 1 [file DataSheet_1.zip › Image 2.PNG]

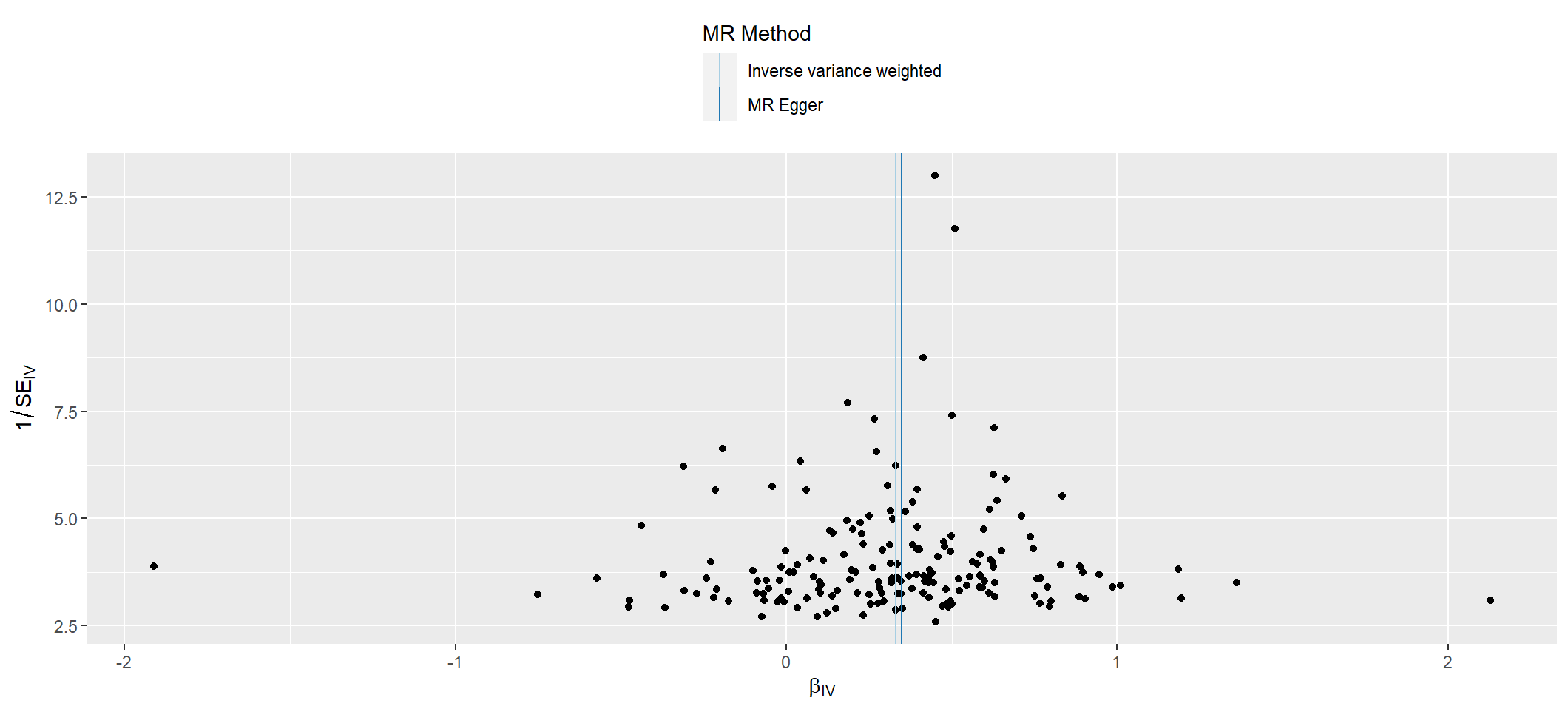

Supplement: Supplementary file 1 [file DataSheet_1.zip › Image 3.PNG]

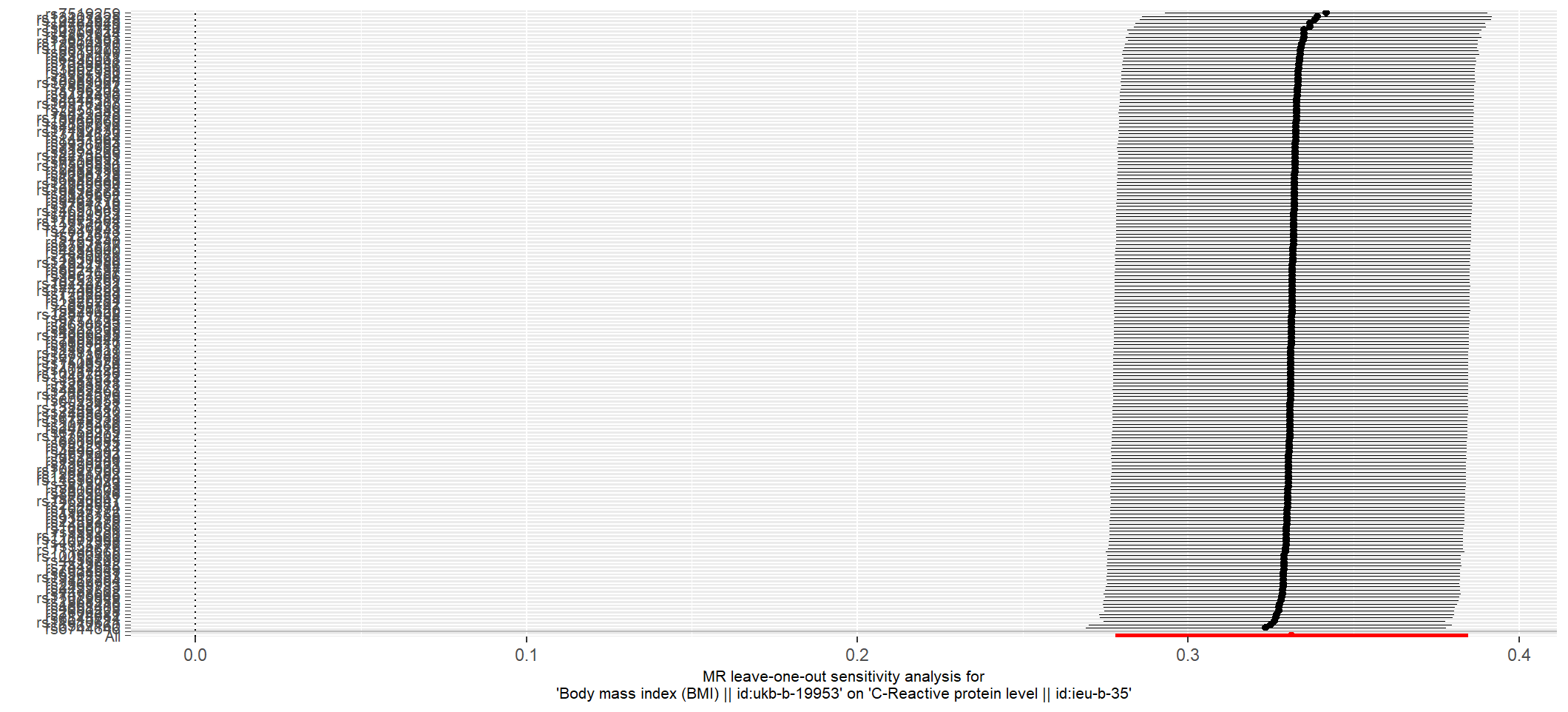

Supplement: Supplementary file 1 [file DataSheet_1.zip › Image 4.PNG]

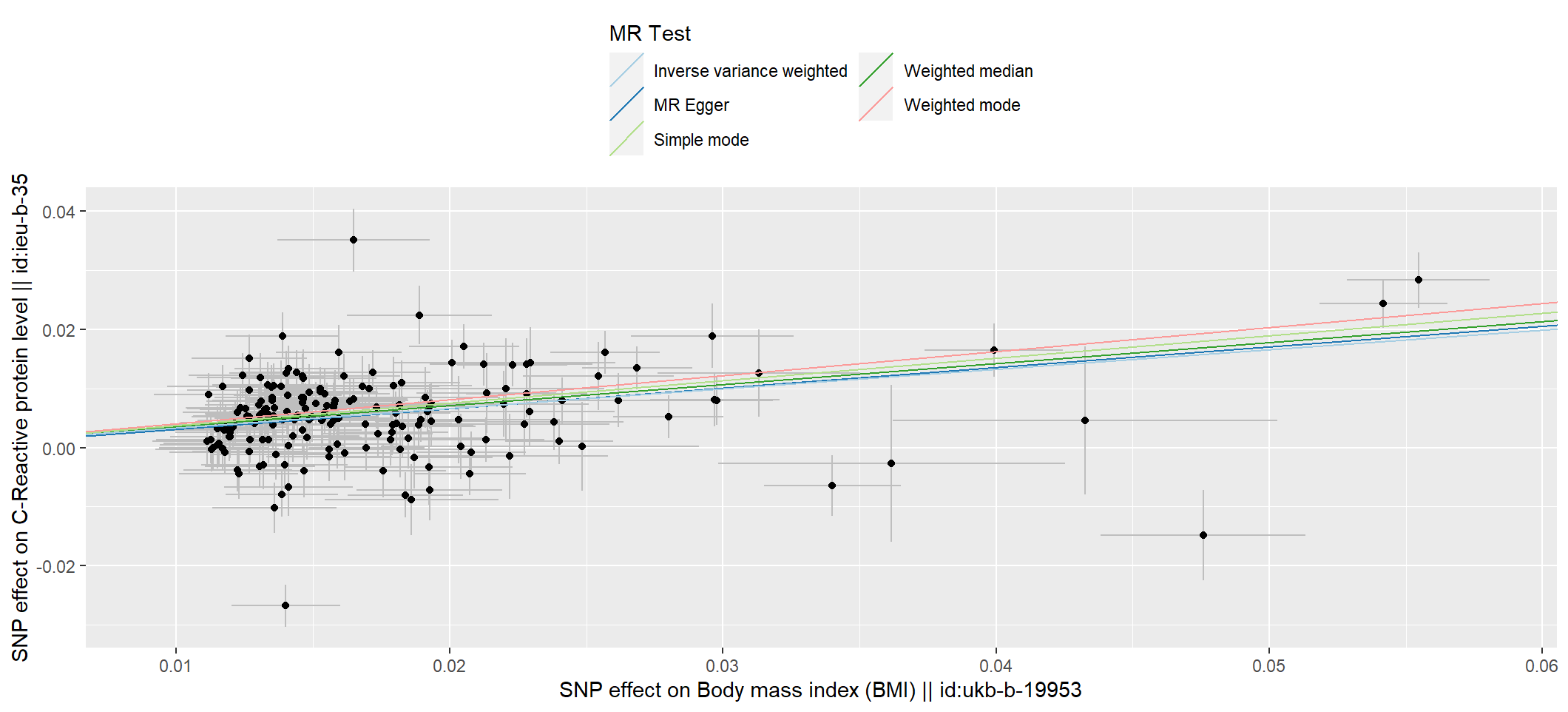

Supplement: Supplementary file 1 [file DataSheet_1.zip › Image 5.PNG]

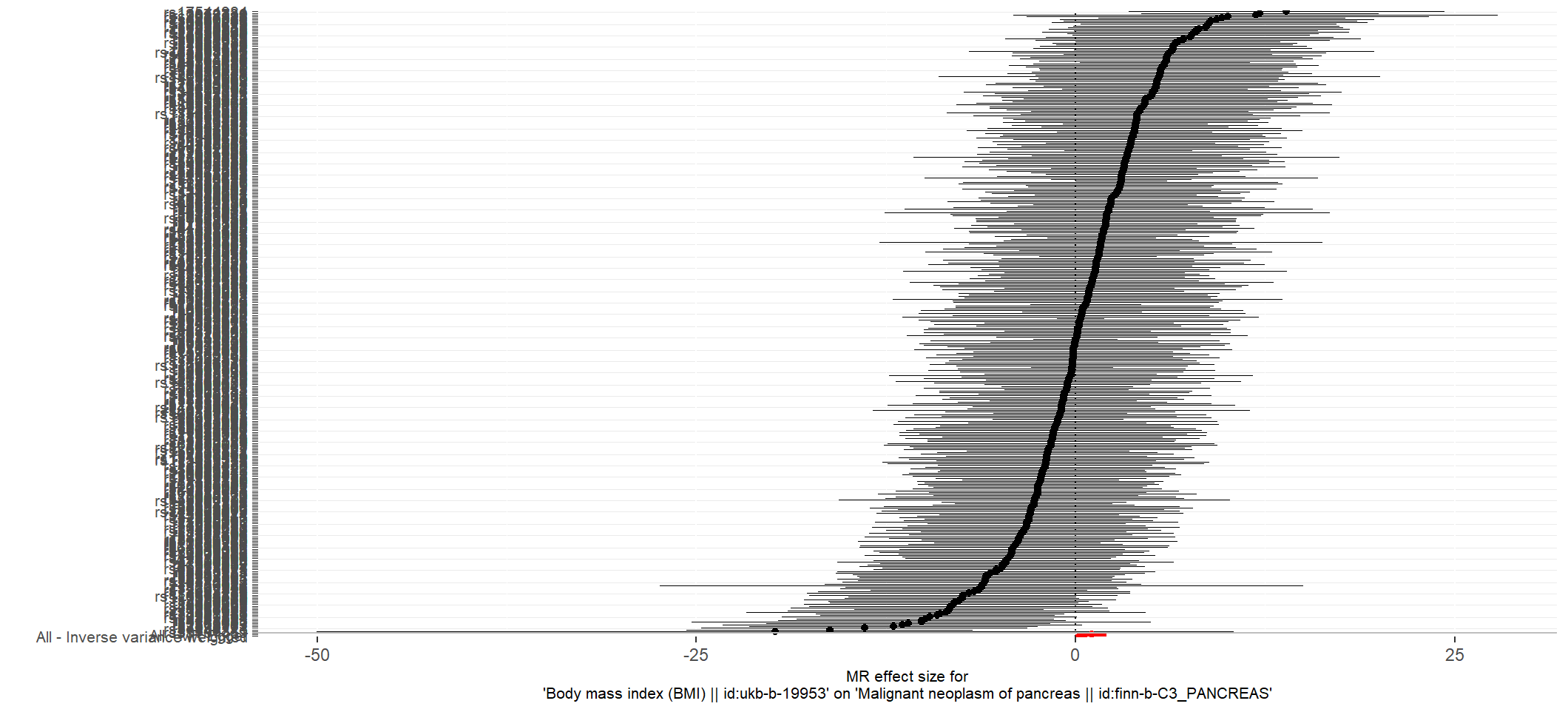

Supplement: Supplementary file 1 [file DataSheet_1.zip › Image 6.PNG]

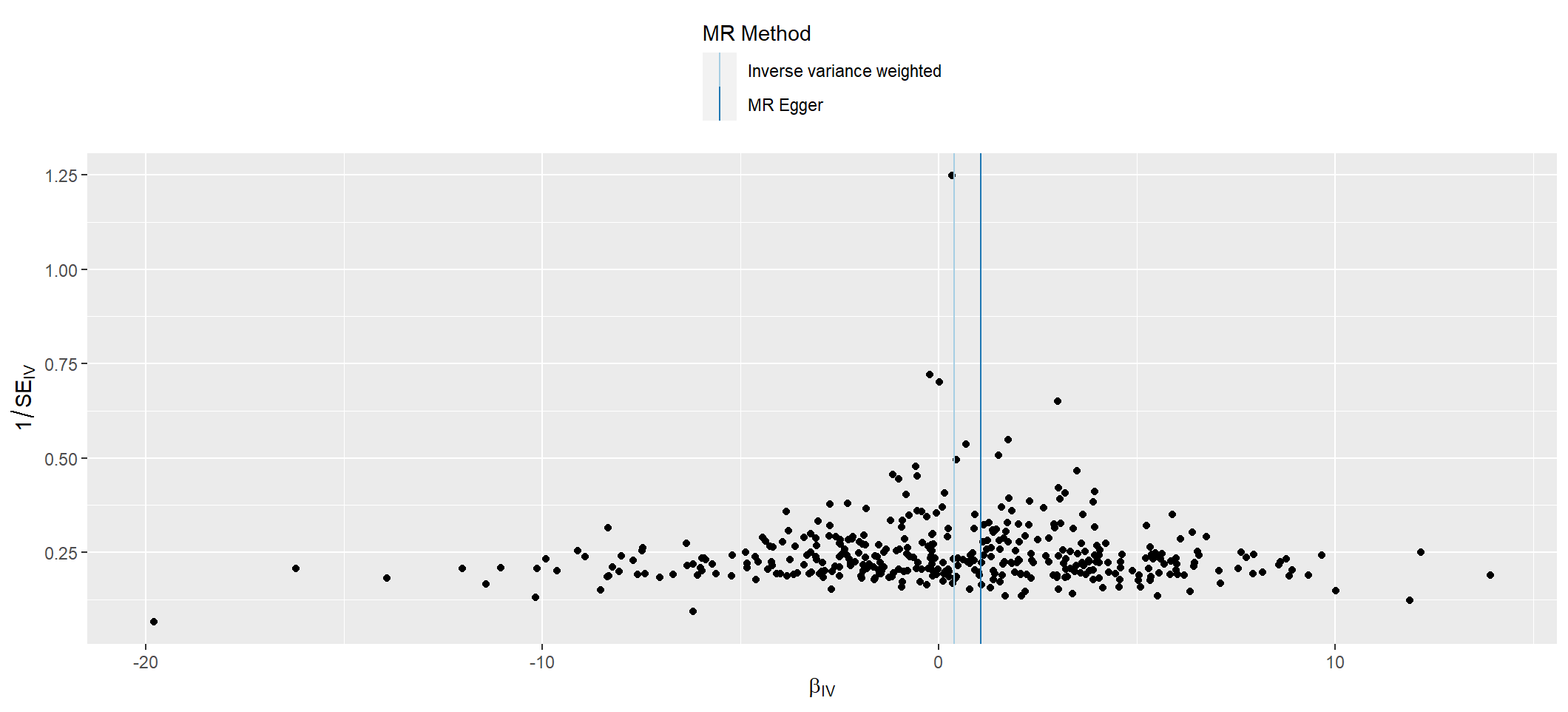

Supplement: Supplementary file 1 [file DataSheet_1.zip › Image 7.PNG]

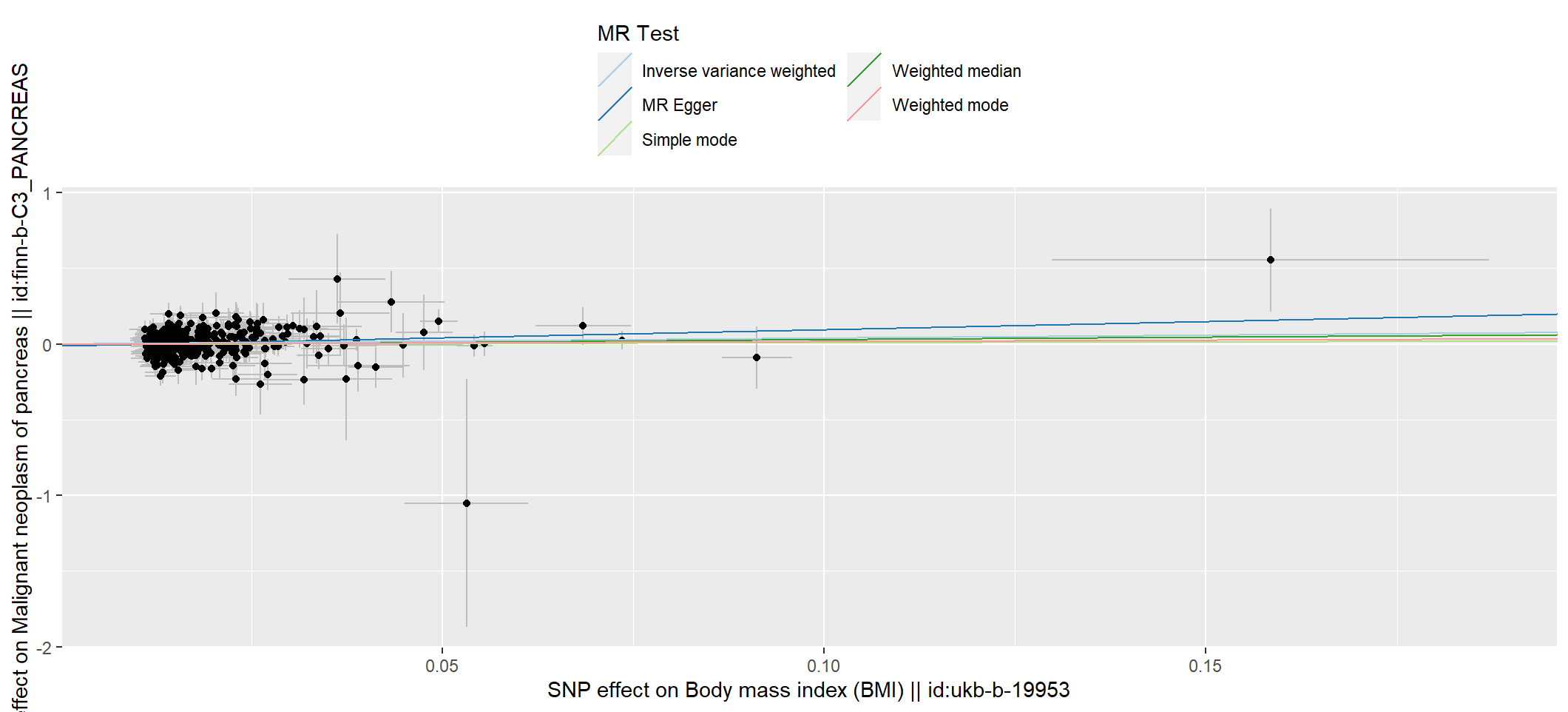

Supplement: Supplementary file 1 [file DataSheet_1.zip › Image 8.PNG]

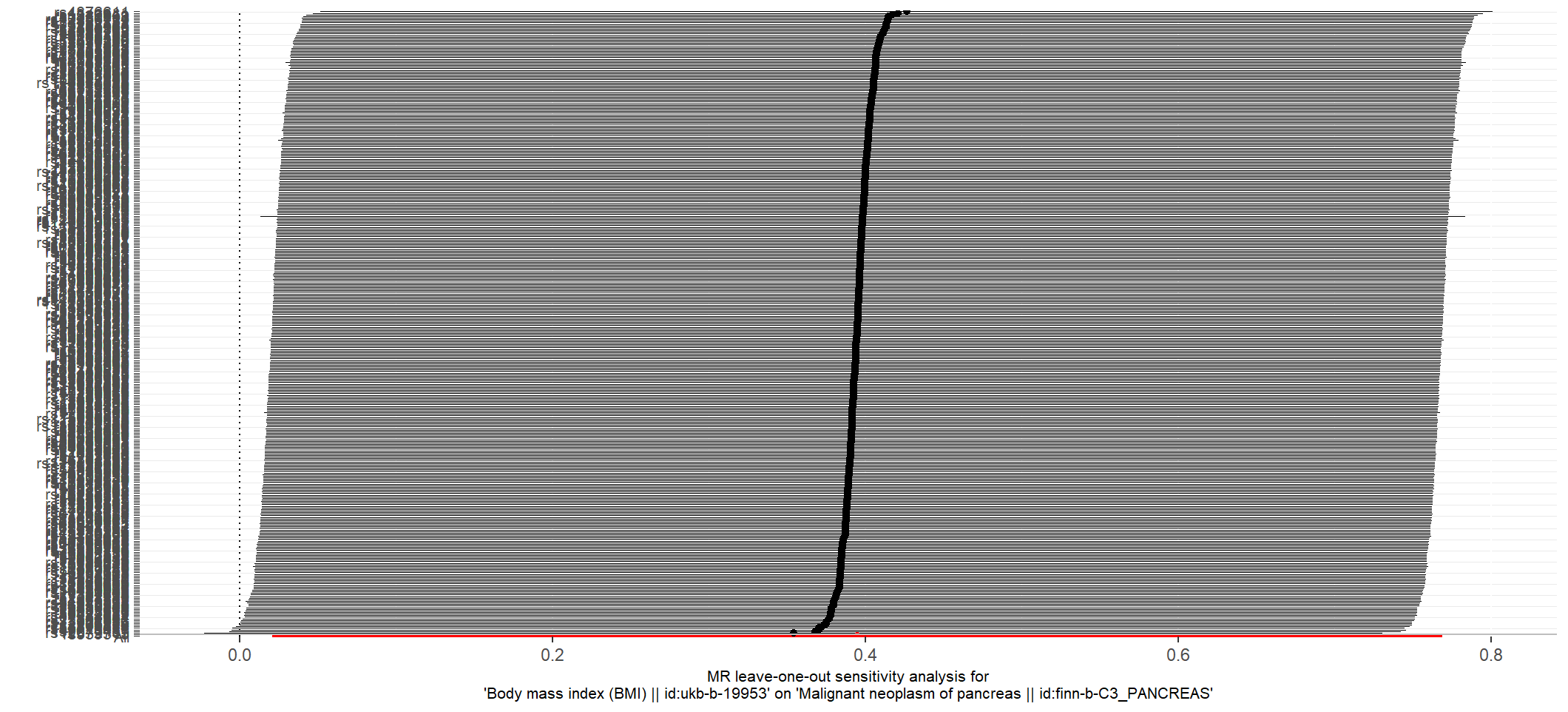

Supplement: Supplementary file 1 [file DataSheet_1.zip › Image 9.PNG]
